# Initial Evaluation of the Effects of Aerosolized Florida Red Tide Toxins (Brevetoxins) in Persons with Asthma

**DOI:** 10.1289/ehp.7500

**Published:** 2005-02-10

**Authors:** Lora E. Fleming, Barbara Kirkpatrick, Lorraine C. Backer, Judy A. Bean, Adam Wanner, Dana Dalpra, Robert Tamer, Julia Zaias, Yung Sung Cheng, Richard Pierce, Jerome Naar, William Abraham, Richard Clark, Yue Zhou, Michael S. Henry, David Johnson, Gayl Van De Bogart, Gregory D. Bossart, Mark Harrington, Daniel G. Baden

**Affiliations:** ^1^National Institute of Environmental Health Sciences Marine and Freshwater Biomedical Sciences Center, University of Miami Rosenstiel School of Marine and Atmospheric Sciences, Miami, Florida, USA;; ^2^University of Miami School of Medicine, Miami, Florida, USA;; ^3^Mote Marine Laboratory, Sarasota, Florida, USA;; ^4^National Center for Environmental Health, Centers for Disease Control and Prevention, Atlanta, Georgia, USA;; ^5^Children’s Hospital Medical Center and University of Cincinnati, Cincinnati, Ohio, USA;; ^6^Lovelace Respiratory Research Institute, Albuquerque, New Mexico, USA;; ^7^Center for Marine Science Research, University of North Carolina at Wilmington, Wilmington, North Carolina, USA;; ^8^Florida Department of Health, Tallahassee, Florida, USA;; ^9^Harbor Branch Oceanographic Institution, Fort Pierce, Florida, USA;; ^10^Twin Cities Hospital, Niceville, Florida, USA

**Keywords:** asthma, brevetoxins, COPD, harmful algal blooms (HABs), *Karenia brevis*, red tides, sensitive populations, spirometry

## Abstract

Florida red tides annually occur in the Gulf of Mexico, resulting from blooms of the marine dinoflagellate *Karenia brevis*. *K. brevis* produces highly potent natural polyether toxins, known as brevetoxins, that activate voltage-sensitive sodium channels. In experimental animals, brevetoxins cause significant bronchoconstriction. A study of persons who visited the beach recreationally found a significant increase in self-reported respiratory symptoms after exposure to aerosolized Florida red tides. Anecdotal reports indicate that persons with underlying respiratory diseases may be particularly susceptible to adverse health effects from these aerosolized toxins. Fifty-nine persons with physician-diagnosed asthma were evaluated for 1 hr before and after going to the beach on days with and without Florida red tide. Study participants were evaluated with a brief symptom questionnaire, nose and throat swabs, and spirometry approved by the National Institute for Occupational Safety and Health. Environmental monitoring, water and air sampling (i.e., *K. brevis*, brevetoxins, and particulate size distribution), and personal monitoring (for toxins) were performed. Brevetoxin concentrations were measured by liquid chromatography mass spectrometry, high-performance liquid chromatography, and a newly developed brevetoxin enzyme-linked immunosorbent assay. Participants were significantly more likely to report respiratory symptoms after Florida red tide exposure. Participants demonstrated small but statistically significant decreases in forced expiratory volume in 1 sec, forced expiratory flow between 25 and 75%, and peak expiratory flow after exposure, particularly those regularly using asthma medications. Similar evaluation during nonexposure periods did not significantly differ. This is the first study to show objectively measurable adverse health effects from exposure to aerosolized Florida red tide toxins in persons with asthma. Future studies will examine the possible chronic effects of these toxins among persons with asthma and other chronic respiratory impairment.

Florida red tides annually occur in the Gulf of Mexico and result from blooms of the marine dinoflagellate *Karenia brevis*. *K. brevis* produces highly potent natural polyether toxins, known as brevetoxins, that activate voltage-sensitive sodium channels. Studies in experimental animals have shown that brevetoxins can cause upper and lower respiratory irritation. The two known clinical entities in humans that are associated with red tide toxins, first characterized in Florida, are acute gastroenteritis with neurologic symptoms after ingestion of contaminated shellfish [i.e., neurotoxic shellfish poisoning (NSP)] and an apparently reversible upper respiratory syndrome after inhalation of the Florida red tide aerosols (; Backer et al. 2003; Baden et al. 1995; Cheng et al. 2005a; Fleming et al. 2001 Fleming et al. 2005; Morris et al. 1991; Music et al. 1973; Pierce et al. 2003; Poli et al. 2000). Anecdotal reports and a prior study of recreational beachgoers have indicated that the inhalation of the aerosols associated with Florida red tide leads to both upper and lower respiratory symptoms, possibly with chronic health implications (Backer et al. 2003; Kirkpatrick et al. 2004).

Respiratory effects from exposure to Florida red tides or pure brevetoxins have been reported in experimental animals. Franz and LeClaire (1989) reported respiratory failure in less than 30 min in guinea pigs exposed intravenously to 0.016 ng/kg of brevetoxin 3. Benson et al. (1999) exposed 12-week-old male F344/Crl BR rats once to 6.6 μg/kg brevetoxin 3 through intratracheal instillation. The researchers concluded that the potential adverse health effects associated with inhaled brevetoxins could extend beyond the reportedly transient respiratory irritation to long-term impacts on asthma. Wells et al. (1984) reported increased airway resistance in Hartley guinea pigs when brevetoxin was inhaled as an aerosol or applied to the nares as nose drops. In unanesthestized asthmatic sheep, intra-tracheal instillation of picogram doses of brevetoxin 3 can cause a significant and rapid increase in airway resistance; this brevetoxin-induced bronchospasm can be effectively blocked by atropine, the mast cell–stabilizing agent cromolyn, the histamine H_1_ antagonist chlorpheniramine, and the β _2_ agonist albuterol (Abraham et al. 2003, 2005; Baden et al. 2005; Benson et al. 2005; Singer et al. 1998). Thus, aerosolized brevetoxins appear to be potent respiratory toxins, involving both cholinergic and histamine-related mechanisms.

Multiple die-offs of marine mammals, particularly the endangered Florida manatee, have been reported in association with Florida red tide and brevetoxins (Bossart et al. 1998). These die-offs probably resulted from exposure to brevetoxins with prolonged inhalation of the red tide toxin aerosol and/or the ingestion of contaminated seawater over several weeks. Necropsies of the dead manatees revealed severe catarrhal rhinitis, pulmonary hemorrhage and edema, and nonsuppurative leptomeningitis, as well as possible chronic hemolytic anemia with multiorgan hemosiderosis and evidence of neurotoxicity.

Few reports have been published about possible adverse health effects associated with exposure to aerosolized red tide toxins in humans. After initial cases in Florida and Texas, Woodcock (1948) reported respiratory irritation during a severe red tide on the west coast of Florida in 1947. The exposure usually occurs on or near beaches during an active red tide bloom. Onshore winds and breaking surf result in the release of the toxins into the water and into the onshore aerosol (Pierce and Kirkpatrick 2001). Collection of marine aerosol along the gulf coast of Florida and the North Carolina Atlantic coast during natural red tide blooms showed that the aerosolized toxins were the same as those in the water and as those identified in the *K. brevis* cultures (Pierce et al. 1990). Aerosolized brevetoxin concentrations, particle size, and exposure level were measured in a recent Florida red tide episode in Texas associated with respiratory symptoms in humans (Cheng et al., 2005a; Pierce et al. 2003). The mass median aerodynamic diameters were 7–9 μm, a relatively large size for inhaled ambient particles.

In humans, inhalation of aerosolized red tide toxins reportedly results in conjunctival irritation, copious catarrhal exudates, rhinorrhea, nonproductive cough, and wheezing (Asai et al. 1982; Backer et al. 2003; Cheng et al., 2005a; Kirkpatrick et al. 2001, 2004; Music et al. 1973 ). The normal population can reportedly rapidly reverse the irritation and wheezing by leaving the beach area or entering an air-conditioned area (Baden 1983; Steidinger and Baden 1984). However, persons with asthma apparently are particularly susceptible to aerosolized Florida red tide and its toxins. In addition to anecdotal reporting, Asai et al. (1982) found that 80% of 15 persons with asthma exposed to red tide aerosol at the beach complained of subsequent asthma attacks. The possible susceptibility of persons with asthma to aerosolized brevetoxins is corroborated by recent investigations with an asthmatic sheep model evaluating the exposure of aerosolized red tide toxins, as discussed above (Abraham et al. 2003, 2005; Singer et al. 1998).

This report presents the initial evaluation of the exposures and effects of aerosolized Florida red tide toxins in persons with prior physician-diagnosed asthma during 1 hr of exposure to a Florida red tide and during 1 hr of nonexposure.

## Materials and Methods

This study was part of the ongoing evaluation of the possible acute and chronic adverse health effects from exposure to aerosolized Florida red tide toxins (brevetoxins) by an interdisciplinary team of researchers from federal, state, private, and local organizations. These studies have received approval from the institutional review boards of the University of Miami School of Medicine, the Centers for Disease Control and Prevention, and the Florida Department of Health. The location for the study was Siesta Beach in Sarasota, Florida.

### Environmental monitoring.

As described by Cheng et al. (2005b) and Pierce et al. (in press), a portable, self-contained weather station was used near the high-volume impactor sampling locations to monitor the air temperature, relative humidity, and wind speed and direction (Complete Weather Station; Davis Instruments, Hayward, CA). The weather station was solar powered, and the data were downloaded into a notebook computer.

Water samples were collected daily in 1-L glass bottles at 0800, 1200, and 1600 hr from the surf zone adjacent to the study’s high-volume air sampler locations. A 20-mL subsample was taken from each bottle and fixed with Utermohl’s solution to provide *K. brevis* cell counts. The remaining water sample was transported to Mote Marine Laboratory and processed for liquid chromatography–mass spectrometry (LC-MS) analysis according to the procedure of Pierce et al. (2003). A sub-sample of each extract was shipped to the University of North Carolina at Wilmington Center for Marine Research for brevetoxin analysis using a new brevetoxin enzyme-linked immunosorbent assay (ELISA) method.

In the laboratory, brevetoxins were extracted by passing the water through a C-18 solid-phase extraction disk under vacuum (Ansys Technologies, Inc., Lake Forest, CA). The C-18 disks then were rinsed with reverse osmosis water to remove any remaining salts and eluted with methanol (Pierce et al. 2003). Brevetoxin analyses were performed by LC-MS using a ThermoFinnigan AqA high-performance liquid chromatography–MS (HPLC-MS; Thermo Electron Corp., Manchester, UK). Mass spectral detection was obtained using an AqA single-quad system scanned from 204 to 1,216 AMU with AqA Max 40 V electrospray. The column was Phenomenex Luna C-18 5Fm 250 × 2 mm (Phenomenex, Torrance, CA); the solvent gradient was 0.3% acetic acid/H_2_O with initial 50:50 acetylnitrile (ACN)/H_2_O to 95:5 ACN/H_2_O over 30 min. The limit of detection (LOD) of the analysis for brevetoxins in seawater was 0.03 μg/L (Cheng et al. 2005b; Pierce et al. in press).

Air samples were collected using three different instruments: high-volume air samplers, high-volume air samplers equipped to capture aerosol particles by size, and personal breathing zone samplers. Brevetoxins were collected in air samples along Siesta Beach. Six high-volume air samplers (TE-5000; Tisch Environmental, Inc., Village of Cleaves, OH) with a single-stage filter were used; three were placed near the surf zone (about 25 m) approximately 100 m apart, and a second row of three was located approximately 50 m from the first row for assessment of aerosolized toxin concentrations over time and space along the beach. Two additional high-volume air samplers with five-stage impactors were deployed in the first row to obtain the distribution of toxin-containing aerosol particle size. Filter samples were collected separately for morning and afternoon time periods (0830–1200 hr and 1230–1600 hr). The impactor samplers were operated from morning to afternoon to collect enough material for analysis. Personal exposure was measured on volunteers wearing a personal sampler (IOM inhalable dust sampler; SKC, Inc., Eighty Four, PA) connected to a battery-operated pump (model 224-PCXR4; SKC, Inc.) while they were at the beach. The personal sampler was placed at the lapel near the breathing zone. A 25-mm glass-fiber filter (type A/E; Pall Life Science, Ann Arbor, MI) was used as the collection substrate. The sampling flow rate was 2 L/min controlled by a rotameter in the sampling pump (Cheng et al. 2005b; Pierce et al., in press).

The high-volume air samplers used to detect aerosolized brevetoxins were fitted with a 20.32 cm × 25.4 cm glass-fiber filter (EPM2000; Whatman, Maidstone, UK). Brevetoxins associated with marine aerosols were recovered from glass-fiber filters by extraction for 12 hr in acetone using a Soxhlet apparatus (Pierce et al. 2003). The extract was then transferred to vials using methanol for LC-MS and ELISA analysis. Brevetoxin recovery from glass-fiber filters was verified by the addition of standard amounts of brevetoxin 2 and brevetoxin 3 to each of three filters that were subsequently processed for LC-MS analyses. Cellulose filters from impactor samplers were cut in half and rolled into a 15-mL polypropylene tube (second half of filter was stored). Ten milliliters of acetone was added to the tube; then the sample was vortexed for 20-sec, sonicated for 2-min, and then placed on a circular rotator (Roto-Torque, low speed-10; Cole-Parmer Instruments, Vernon, IL) for 20-min. The 10 mL of extract then was evaporated under a gentle stream of nitrogen to approximately 100 μL, vortexed for 5 sec to rehomogenize the extract, and recombined with 50:50 methanol:purified water to the final analysis volume (typically 200 μL) (Cheng et al. 2005a; Pierce et al., in press). The samples then were analyzed for brevetoxins by an LC-MS technique using an HPLC (SIL-DAD vp; Shimadzu Co., Kyoto, Japan) coupled with the API 365 MS/MS (Applied Biosystems Inc., Foster City, CA) (Cheng et al. 2005a; Pierce et al., in press). The LOD for the analysis of impactor samples was 0.01 ng/m^3^.

Concentrations of brevetoxins on portions of the ambient air filters and the personal breathing zone filters, as well as the nasal and throat swabs, were also analyzed by a recently developed competitive ELISA (Naar et al. 2002). Air filters (personnel and aliquots of environmental air filters) were sonicated for 15 min in ELISA buffer (0.1 M phosphate-buffered saline, pH 7.4, 0.5% gelatin, 0.1% Tween 20). Nose and throat swabs were sonicated for 45 min in ELISA buffer. The sonicated material was analyzed directly according to the brevetoxin ELISA protocol (Naar et al. 2002). The limit of quantification of the brevetoxins using the ELISA was 0.6 ng/sample (air filter and/or nasal/throat swab).

### Adverse human health effects.

Persons at least 12 years of age who reported a physician’s diagnosis of asthma were recruited as study participants in Sarasota, Florida, an area with a history of annual Florida red tides. All participants who gave informed consent to participate walked on the beach once during a Florida red tide and also walked once when no Florida red tide was present. Participants were instructed to maintain their daily regime for asthma control. They were asked to spend a minimum of 1 hr at the beach in areas where environmental monitoring was ongoing and were told they could leave the beach at any time if they felt uncomfortable or symptomatic and could freely use any personal medications. Study activities included the pre- and postexposure questionnaires, swab sampling, and spirometry, as well as carrying a personal air monitor while at the beach.

Questionnaires were administered upon enrollment and then before and after the participants visited the beach. The questionnaires collected information about demographics, baseline pulmonary health history, prior experience with Florida red tide, medications, potential confounders, and symptoms. In addition to asking about expected common respiratory symptoms, the questionnaire asked about diarrhea to detect overreporting bias, because diarrhea was not expected to be associated with exposure to aerosolized Florida red tide (vs. ingestion exposure resulting in NSP). For the purposes of analysis, age groups were evaluated as tertiles (< 18 years, 18–60 years, > 60 years). Geographic proximity of residence to the beach also was explored; proximity was defined as residence on a barrier island or along Sarasota Bay (i.e., within approximately 1 mile of the seashore). The investigators considered two surrogate measures of asthma severity: above and below the unexposed period study population mean forced expiratory volume in 1 sec (FEV_1_) and the use of asthma medications (predominantly β _2_ agonists) within 12 hr before going to the beach. However, the mean FEV_1_ before the unexposed beach visit was not considered to be a good measure of disease severity and was not used: no untreated baseline was obtained before the unexposed beach visit, and in the study population, persons who used asthma medication within 12 hr before going to the beach, were more likely to have an FEV_1_ value greater than the mean FEV_1_ from the unexposed period (data not shown). Therefore, only the use of asthma medications within 12 hr before going to the beach was used as a surrogate for increased asthma severity.

Spirometry tests were performed using a portable OMI2000 10-L dry-rolling-seal volume spirometer (Occupational Marketing, Inc., Houston, TX) by personnel trained according to the standards of the National Institute for Occupational Safety and Health (NIOSH 1997). The spirometric values of interest were FEV_1_, forced vital capacity (FVC), forced expiratory flow between 25 and 75% (FEF_25–75_, peak expiratory flow (PEF), and FEV_1_/FVC percentage. For the purposes of this study, each participant served as his or her own spirometry control (i.e., pre-exposure/postexposure); all study participants had at least three reproducible spirograms before and after visiting the beach. The data were considered adequate if they conformed to standard guidelines for the collection and interpretation of spirometry measurements (American Thoracic Society 1991, 1995; Hankinson et al. 1999).

As an effect biomarker of inflammation, nose and throat swabs were collected from the study participants before and after they went to the beach. Samples were obtained by gently wiping the nose or throat with a cotton-tipped swab, smearing the material onto duplicate microscope slides, and fixing with a cytologic adhesive spray (Spray-Cyte; Becton Dickinson, Sparks, MD). One slide from each pair was stained using Diff Quik (Dade Behring Inc., Newark, DE) for cytologic evaluation of epithelial and inflammatory cells. The inflammatory response was characterized according to cellularity and the percentage of neutrophils and chronic inflammatory cells (e.g., macrophages, lymphocytes, and plasma cells). In addition, protein transudation and amount of fibrin present were evaluated because increasingly permeable cell membranes, a key event in the inflammatory process, lead to protein transudation as proteins leak out of the cells. As described above, brevetoxin levels also were analyzed by a newly developed brevetoxin ELISA (Naar et al. 2002) on the nasal and throat swabs as an exposure biomarker.

### Statistics.

A study database was created in Microsoft Access. Descriptive and other statistical analyses were performed using SAS statistical software (version 8.03; SAS Institute Inc., Cary, NC). Statistical hypothesis testing was performed using the paired *t*-test for continuous data and the McNemar’s test for categorical data (Kleinbaum et al. 1982) to compare pre- and postexposure data. The number of persons reporting no symptom before going on the beach but reporting the particular symptom after exposure was compared with the number who reported no symptoms before and after their beach walk. Because this was an initial evaluation of the possible human adverse health effects of Florida red tide airborne toxin exposure, it was considered hypothesis generating. Therefore, multiple comparisons were not adjusted for in the level of statistical significance.

## Results

The results contrast the environmental monitoring and human adverse health effects evaluations collected during 3 days when study participants were exposed to the Florida red tide aerosol (exposed sampling period, March 2003) with similar data collected during 3 days when no Florida red tide occurred (unexposed sampling period, January 2003).

### Environmental data.

Environmental sampling confirmed the presence of *K. brevis* and brevetoxins in the water and air during the Florida red tide exposed period, and the lack of significant toxin and organism levels in the water and air during the Florida red tide unexposed evaluation (Table 1). Surf water samples from the unexposed period exhibited *K. brevis* cell counts ranging from none detected (< 1,000 cells/L) to a high of 6,000 cells/L on 17 January 2003, diminishing to < 1,000 cells/L on 18 January. During this time, water samples contained low concentrations of brevetoxins, ranging from none detected (< 0.05 μg/L) to 2.0 μg/L for samples collected on both days. High-volume air samplers recovered only trace amounts of brevetoxins from the air on 17 January, with none detected (< 0.5 ng/m^3^) on 18 January.

Water samples collected during the Florida red tide exposed period showed moderate to high concentrations of *K. brevis* cells (daily mean ± standard deviation) on the first day of the exposed study, 29 March 2003 (181,000 ± 131,000 cells/L). Cell concentrations increased on the second day of exposed study on 30 March (764,000 ± 264,000 cells/L) and remained high through 31 March morning (236,000 ± 69,000 cells/L), then diminishing rapidly by the 1200 hr sample collection (22,000 ± 11,000 cells/L). The mean total brevetoxin concentration in water samples on 29 March was 3.4 ± 1.9 μg/L throughout the day. Toxin concentrations in water were higher on 30 March, with a mean and standard deviation of 14 ± 8 μg/L, diminishing again to 3.3 ± 3.7 μg/L on 31 March.

In addition to temperature and relative humidity, wind speed and wind direction were measured. Both wind speed and wind direction are essential in the production and transport of the red tide aerosol to the beach. During the unexposed Florida red tide period, wind direction during the sampling was offshore. This and low brevetoxin concentration in water resulted in only trace or undetectable amounts of brevetoxin concentration in the air. During the first 2 days of the exposed Florida red tide period, the wind direction was partially onshore with medium to high concentrations of brevetoxins in water. These environmental conditions produced medium levels of brevetoxins in the air. On 31 March, the last day of the exposed Florida red tide period, the wind direction changed to offshore wind, and the air concentration of brevetoxins was much lower.

By LC-MS analyses, concentrations of brevetoxins in the air samples were found throughout the first day of the study, 29 March, with an overall daily mean and standard deviation of 37 ± 18 ng/m^3^. Samples collected near the surf did not differ from those collected 50 m up the beach, probably because of the strong winds rapidly dispersing the toxins, providing uniform exposure over the beach study area. Aerosolized brevetoxin concentrations in the ambient air diminished on 30 March to < 1/10th that observed on the first day of the exposed study, with none detected in air samples on 31 March, even though the cell counts and brevetoxin concentrations remained high in the water. This probably occurred because of a shift in wind direction from onshore to alongshore and offshore.

ELISA analyses detected no brevetoxins on either the swabs or the personal monitoring during the unexposed study period (January 2003). In the exposed study period (March 2003), brevetoxins were detected in seawater, environmental and personnel air monitoring filters, and nose swabs of some of the participants by ELISA. However, in initial experimental analysis, the presence of toxins in nose swabs did not appear to be simply correlated with the amount of toxin that participants were exposed to in their breathing zone (i.e., the toxin levels measured on personnel air monitoring filters). During the other 2 days of the Florida red tide exposed period, toxin levels in the sea spray at the beach were too low to be detected on air personnel filters and/or on nose swabs with the brevetoxin ELISA.

The particle size distribution from the impactor sample could be represented by a log-normal distribution. The mass median aerodynamic diameter was 6.54 ± 1.34 μm, with a geometric standard deviation of 1.73 ± 0.05 μm.

### Adverse health effects.

Of the 130 persons initially enrolled in the study of sensitive sub-populations, 59 who had asthma participated in study activities during both an unexposed (January 2003) and an exposed (March 2003) study period evaluation. Their mean age was 35.8 ± 18.7 years (range, 12–69 years); most were white non-Hispanic women (Table 2). This population was relatively healthy, with very few current smokers [5 (8.5%)]; however, 11 (19.6%) had been hospitalized at least once in the past year for pulmonary reasons. Most (93.2%) of these participants reported variable use of asthma medications (predominantly β _2_ agonists) and had experienced a Florida red tide with reported symptoms (82.3%).

During the unexposed period, the 59 participants who had asthma experienced neither significant respiratory impairment nor development of symptoms after being at the beach for 1 hr. The participants were significantly more likely to report symptoms and significantly more likely to have a measurable respiratory impairment on spirometry after going to the beach for 1 hr during a Florida red tide exposure (Table 3), although there was considerable variation in the respiratory function during the January 2003 unexposed period. The significant symptoms reported only during active exposure to Florida red tide included respiratory complaints of cough (*p* < 0.01), wheezing (*p* < 0.03), and chest tightness (*p* < 0.02), as well as throat (*p* < 0.02) and eye irritation (*p* < 0.01). Participants did not report diarrhea (*p* = 1.00). Statistically significant decreases in respiratory function during the Florida red tide exposed period were measured for the FEV_1_ (38.0 ± 118.0 mL; *p* < 0.02) and the FEF_25–75_ (95.0 ± 296.0 mL/sec; *p* < 0.02) with significant change from preexposure. No significant changes in the FVC or FEV_1_/FVC percentage were seen.

The association between symptoms and change in FEV_1_ during Florida red tide exposure was evaluated as *a*) greater or less than the mean change in FEV_1_ (i.e., preexposure minus postexposure FEV_1_) and *b*) as a positive change (i.e., preexposure > postexposure FEV_1_) vs. negative change (i.e., preexposure < post-exposure FEV_1_). Participants with a decrease in FEV_1_ after exposure to Florida red tide reported statistically significant increased coughing (*p* < 0.04) and borderline increased difficulty breathing (*p* < 0.06) and chest heaviness (*p* < 0.06). However, asthmatics with an increase in FEV_1_ after exposure to Florida red tide reported a statistically significant increased chest heaviness (*p* < 0.01) but no other respiratory symptoms. Of note, reporting a cough (not wheezing) after going to the beach was associated with statistically significant decreases in the PEF (*p* < 0.03) for asthmatics only during the Florida red tide exposed period.

Because the participants lived in an area with annual and often continuous Florida red tides, there was the possibility that their decreased respiratory function before the study beach walk existed because of some prior exposure (e.g., a prior Florida red tide exposure). This possibility was examined by comparing the pulmonary function before the beach walk during the unexposed time period with the pulmonary function before the beach walk during the Florida red tide exposed period. No significant differences were found between FEV_1_, FEF_25–75_, PEF, FVC, or FEV_1_/FVC values before the unexposed beach walk and those before the exposed beach walk. In fact, the mean pulmonary function was slightly better before the beach walk during the Florida red tide exposed period than it was before the unexposed period (data not shown).

Asthmatics who lived away (> 1 mile) from the seashore were significantly more likely to report respiratory symptoms after going to the beach during the Florida red tide exposed period, whereas those who lived close (i.e., within ≤1 mile) to the seashore had no statistically significant increase in reported symptoms. Asthmatics who lived far from the beach also were significantly more likely to experience a decrease in FEV_1_ (48.0 ± 122.3 mL; *p* < 0.01) and FEF_25–75_ (104.1 ± 254.7 mL/sec; *p* < 0.009) with significant change after beach exposure to Florida red tide. Respiratory function did not differ in asthmatics who lived close to the beach during the Florida red tide exposed period.

The day with the highest Florida red tide exposure by air environmental monitoring (29 March) was examined, although only 29 asthmatics were evaluated on that day. Reported symptoms and pulmonary function did not change significantly during their unexposed evaluations. During the Florida red tide exposed period, these participants reported significantly more cough (*p* < 0.02) after returning from the beach, and there was a statistically significant decrease in FEF_25–75_ (116.9 ± 272.9 mL/sec; *p* < 0.04).

Additional analyses determined whether other factors such as age, gender, and severity of illness were important with regard to reported symptoms and spirometry before and after going to the beach (data not shown). Only the 18- to 60-year-old participants had statistically significant increases in reported respiratory symptoms during the Florida red tide exposed period, but not in the unexposed period. Age was not associated with any significant changes in spirometry in the unexposed period, but during the Florida red tide exposed period, only asthmatics 18–60 years of age had statistically significant decreases in FEV_1_ (*p* < 0.04), FEF_25–75_ (*p* < 0.03), and PEF (*p* < 0.05) after going to the beach. No significant differences for the symptoms were reported by gender during the unexposed period; during the Florida red tide exposed period, female asthmatics reported significantly more respiratory symptoms after going to the beach. During the exposed period, there were statistically significant decreases in FEV_1_ (*p* < 0.009) and FEF_25–75_ (*p* < 0.05) for female asthmatics, as well as statistically significant decreases in FEV_1_ (*p* < 0.02) during the unexposed period; male asthmatics had no statistically significant changes during the Florida red tide exposed or unexposed periods.

To explore asthma severity, the surrogate measure was used to compare those asthmatics who did and did not use asthma medications within the 12 hr before going to the beach. More persons [42 (58%)] reported using asthma medication within 12 hr before going to the beach during the exposed to Florida red tide time period than during the unexposed time period [30 (42%)]. The use of asthma medications within 12 hr before going to the beach was associated with statistically significant increased report of cough (*p* < 0.004) and chest heaviness (*p* < 0.04) after going to the beach during the Florida red tide exposed period only. The reported use of asthma medications 12 hr before the beach walk was associated with no changes in the unexposed period, but with statistically significant changes in the FEV_1_ (45.0 ± 100.1 mL; *p* < 0.02), FEF_25–75_ (120.0 ± 299.3 mL/sec; *p* < 0.03), and PEF (160.0 ± 435.7 mL/sec; *p* < 0.04) during the Florida red tide exposed period. Asthmatic females [22/34 (65%)] were more likely to report using medication before going to the beach compared with males [10/25 (40%)]; female asthmatics using medications experienced statistically significant decreases in their pulmonary function during the Florida red tide exposed period, particularly FEV_1_ (78.0 ± 93.6 mL; *p* < 0.0008) and FEF_25–75_ (166.4 ± 423.7 mL/sec; *p* < 0.04). Asthmatics of both genders who lived far from the beach were more likely to report taking medication before going to the beach [25 of 44 (57%)] compared with those who lived close to the beach [7 of 15 (47%)]; these same asthmatics were more likely to experience statistically significant decreases in their pulmonary function during the Florida red tide exposed period, particularly FEV_1_ (57.6 ± 104.9 mL; *p* < 0.01) and FEF_25–75_ (132.0 ± 273.6 L/sec; *p* < 0.02). The seven asthmatics taking medication who lived near the beach experienced a statistically significant decrease in PEF (430.0 ± 394.0 mL; *p* < 0.03) during the Florida red tide exposure period.

### Swabs.

The 59 asthmatics were sampled by nasal and throat swabs both when unexposed (January 2003) and exposed to Florida red tide (March 2003). The Florida red tide exposed swab data were compared with the unexposed swab data. Several parameters were evaluated (i.e., inflammation between pre- and post-beach walk samples, protein transudation, amount of fibrin present in the sample, and percentage of reactive cells). These parameters were all higher, although not statistically significant (chi square, 0.1 > *p* > 0.05), on the second exposed day (30 March) than throughout the unexposed period when no Florida red tide was present. Analyses among the 3 days of Florida red tide exposure indicated that the swab samples on the second exposed day (30 March) showed the greatest increase in inflammatory response within the day (i.e., postexposure samples contained a greater inflammation than preexposure sample); the first exposed day (29 March) was intermediate, and the last exposed day (31 March) had the least increase in inflammation. On the second exposed day, more (although again not statistically significant) protein and fibrin and a larger percentage of reactive epithelial cells were found (chi square, 0.1 > *p* > 0.05) in the samples than on the other days.

## Discussion

This is the first study to demonstrate measurable adverse health effects, both in terms of reported symptoms and objectively measured respiratory decreases, from exposure to aerosolized Florida red tide toxins in persons with asthma. In addition, this study documents the water and air exposures to the aerosolized Florida red tide toxins associated with these adverse health effects. This study shows that just visiting the beach did not appear to adversely affect health for persons with asthma during periods of no red tide, despite relatively low temperatures and strong winds; in the past, these environmental factors have been associated with increased bronchoconstriction among asthmatics (Koh and Choi 2002). In fact, additional analysis of data collected on a subsequent unexposed warmer and more humid day in May 2004 (data not shown) illustrated that there was some effect of the cold temperatures in the January 2003 nonexposure period on the lung function of the asthmatics; however, the March 2003 Florida red tide toxin exposure caused substantial significant respiratory changes in the asthmatics.

In this preliminary study, the asthmatics who appeared to be at greatest risk for a statistically significant respiratory decrease after exposure to Florida red tide were those who chronically used medications. As discussed above, although precisely defining the asthma severity is not possible without an untreated unexposed baseline assessment of all the asthmatics, the most severe asthmatics in this study population are probably those who reported regular use of medications within 12 hr of going to the beach for the study. These more severe asthmatics had the most significant decreases in respiratory function during Florida red tide exposure among all the study participants. Furthermore, the effects of other possible factors (i.e., age, sex, and residence proximity to the shore) were less important when the data were stratified by use of medication. Therefore, the results suggest that the more severe asthmatics were the most sensitive to Florida red tide toxin exposure.

Preliminary work with an asthmatic sheep model has indicated that pretreatment with regularly used asthma medications (i.e., cromolyn, albuterol, and even antihistamines) minimize the respiratory depressive effects of brevetoxins and of Florida red tide aerosol. Therefore, the respiratory decreases in participants with more severe asthma in this study might have actually been much greater if they had not premedicated.

The brevetoxin dose to which the study participants were exposed was relatively low, with an average ambient air concentration on the beach even during the highest exposure day (29 March) of 36.67 ± 17.54 ng/m^3^. In a prior study of recreational beachgoers by these investigators, this was considered “moderate” exposure compared with a “high” exposure day with up to 108 ng/m^3^ of brevetoxin in the air (Backer et al. 2003, 2005). If an average adult at rest breathes in about 6 L of air per minute (Guyton 1981), then persons visiting the beaches during this study on the highest exposure day inhaled approximately 12.9 ng of brevetoxin each hour, or an inhaled dose of 0.18 ng/kg (assuming an average weight of 70 kg) each hour. Franz and LeClaire (1989) reported respiratory failure in < 3 min in guinea pigs exposed intravenously to 0.016 ng/kg brevetoxin 3, and Benson et al. (1999) exposed 12-week-old male F344/Crl BR rats to a single dose of 6.6 μg/kg brevetoxin 3 through intra-tracheal instillation resulted in systemic distribution of brevetoxin 3 lasting more than 7 days postexposure. Singer et al. (1998) and Abraham et al. (2003, 2005) have found that intratracheal administration of picogram doses of both brevetoxins and aerosolized Florida red tide samples caused significant respiratory depression in asthmatic sheep. Therefore, aerosolized Florida red tide and brevetoxins appear to be significant respiratory toxins at very low exposures in both humans and animals.

The physiologic impact of exposure to the Florida red tide aerosol depends on the mass and chemical characteristics of the inhaled particles. In a similar study of red tide aerosols conducted in Texas (Cheng et al. 2005a) and in the present study (Cheng et al. 2005b; Pierce et al., in press), the particles containing brevetoxin were 2.9–15 μm in mass median aerodynamic diameter. Inhaled particles of this size would be deposited primarily in the upper respiratory tract (Schlesinger 1985); subsequent respiratory irritation could result from the impact of the particles themselves and/or from the toxins associated with the particles. In the present study, the reported respiratory irritation and the measured decreases in respiratory function were caused by exposure to aerosolized Florida red tide and brevetoxins; in general, the study participants did not report symptoms or have measured respiratory decreases during nonexposure to Florida red tide, but they did when brevetoxins were measured in the environmental air sampling, as well as in their noses, using the newly developed ELISA for brevetoxins.

Possible chronic effects from exposure to aerosolized Florida red tide and brevetoxins may occur and should be evaluated. The swab inflammatory data suggest a possible delayed increased effect among the sensitive subpopulation after the beginning of red tide exposure. Anecdotally, several of the asthmatic participants have reported delayed effects after their Florida red tide beach exposures. This is further supported by another study (Quirino et al. 2004) using Florida Poison Information Center data to compare persons calling with Florida red tide–associated symptoms with unexposed control callers. Callers with Florida red tide exposure reported significantly more respiratory symptoms at the time of exposure, and a significantly longer duration of these symptoms (12.84 ± 25.35 days duration of symptoms compared with 2 ± 1.41 days for the unexposed callers). Also, callers exposed to Florida red tide were significantly more likely than unexposed callers to report seeking medical care (an elevated relative risk of 3.00; *p* < 0.025).

### Study limitations and strengths.

This study has several limitations. First, exposure to aerosolized Florida red tide is difficult to assess. It is a natural event with significant variation over time and space caused by the ongoing effects of seawater concentrations, wind direction and speed, and other environmental factors. Furthermore, the aerosol is a mixture of seawater and salt, various brevetoxins, cellular particles, and other substances as yet to be defined. For example, the study investigators have discovered that *K. brevis* produces a natural inhibitor of brevetoxin, known as brevenol; brevenol was measured during the March 2003 study period on the environmental air samplers (Bourdelais et al. 2004; Cheng et al. 2005b; Pierce et al., in press; Purkerson-Parker et al. 2000). The exact constituents of Florida red tide aerosols and their individual and combined effects on humans and other animals need further evaluation.

This study took place in an area with annual Florida red tide exposure. Defining a complete unexposed period was not possible because there were *K. brevis* cells in the waters along the beach study site even during the “unexposed” period. Furthermore, the participants were all residents of this area, and many had a history of Florida red tide exposure. Therefore, these participants may have experienced intermittent Florida red tide aerosol exposure unmeasured by the investigators before, after, and during the two study periods. In addition, residents of this geographic region may have adapted to chronic Florida red tide aerosol exposure.

Techniques to measure human exposure and subsequent adverse health effects from exposure to aerosolized Florida red tide toxins are currently under development. The newly developed brevetoxin ELISA, as well as LC-MS and HPLC, were close to their LODs even during the Florida red tide exposure period; this was particularly true for the relatively low-flow personal air monitoring and for the swabs. The use of throat and nose swabs to evaluate inflammatory change is also under development. Nevertheless, these methodologies offer the possibility of quantitative and objective measurement with both exposure and effect biomarkers of brevetoxins in humans in the future, in addition to the more traditional exposure assessment (i.e., water, environmental air, personal air) and health effect assessment (i.e., questionnaire and spirometry).

In this initial study, the asthmatic participants were evaluated without Florida red tide exposure but not without the use of their medications. This is important for several reasons. Preliminary data using an asthmatic sheep animal model have shown that pretreatment with commonly used asthma medications (i.e., albuterol, atropine, cromolyn, and even antihistamines) can minimize the effects of brevetoxins and of Florida red tide aerosols on respiratory function (Abraham et al. 2005). Therefore, the ongoing use of asthma medications among the participants with more severe asthma may have decreased the physiologic effects of Florida red tide exposure in the most sensitive subpopulation.

In addition to the advantage of employing the objective effect measure of spirometry, this study used a methodology in which each subject served as his or her own control with regards to all effect measures, before and after 1 hr of beach exposure as well as during Florida red tide exposed and unexposed periods. This methodology has been used extensively looking at cross-shift and at longitudinal changes in respiratory function (Anees 2003; Chan-Yeung 2000; Eisen et al. 1997; Hnizdo et al. 1999; Skogstad et al. 2002; Waters et al. 2003) in occupational studies of respiratory toxins, as well as in environmental air pollution studies (Desqueyroux et al. 2002).

The strengths of this preliminary study of sensitive population and exposure to aerosolized Florida red tide outweigh its limitations. This study integrated extensive environmental assessment with evaluation of adverse human health effects. It is the first study to objectively measure both exposure and adverse health effects in a relatively large population of persons with underlying respiratory disease. Finally, although small, the objectively measured respiratory decreases were statistically significant and correlated with the both the environmental assessment and with the self-reported respiratory symptoms.

The investigators plan to evaluate the possible chronic effects of exposure to aerosolized Florida red tide toxins among the sensitive subpopulations. Not all persons with asthma may be equally sensitive to toxin exposures. In addition, evaluation of possible therapeutic interventions using animal models, as well as controlled trials in humans, needs to be explored.

## Figures and Tables

**Table 1:** Environmental conditions and concentrations of brevetoxins in water and air samples during the unexposed and exposed sampling periods.

				Predominant wind direction (% onshore)			Brevetoxin	
Date	Temperature (°C)	Humidity (%)	Average wind speed (km/hr)		*K. brevis* (cells/L)	Seawater (μg/L)	Air (ng/m^3^)
Unexposed							
17 January 2003	12.2 ± 1.6	68 ± 5	25.6 ± 3.4	Offshore (1%)	2,400 ± 1400	< LOD	< LOD
18 January 2003	8.3 ± 1.6	47 ± 5	10.9 ± 3.7	Offshore (4%)	< LOD	< LOD	< LOD
19 January 2003	13.3 ± 1.1	53 ± 7	12.4 ± 4.0	Offshore (2%)	< LOD	< LOD	< LOD
Exposed							
29 March 2003	24.4 ± 0.5	83 ± 4	10.5 ± 5.4	Partly onshore (58%)	180,600 ± 131,000	3.44 ± 1.93	36.57 ± 17.51
30 March 2003	18.9 ± 2.2	84 ± 6	24.9 ± 6.0	Partly onshore (44%)	764,400 ± 263,700	14.01 ± 8.06	3.71 ± 2.63
31 March 2003	12.8 ± 1.1	32 ± 12	22.7 ± 2.6	Offshore (0%)	96,300 ± 86,400	3.31 ± 3.74	< LOD

**Table 2:** Demographics of 59 physician-diagnosed asthmatic study participants.*^a^*

Variable	Asthmatics [*n* (%)]
*n*	59
Age ± SD (range in years)	35.8 ± 18.7 (12.0–69.0)
Female	34 (57.6%)
Race–ethnicity	
White (%)	58 (98.3%)
Hispanic (%)	2 (3.4%)
Years with diagnosis ± SD	18.2 ± 14.9
Using asthma medications currently*^b^*	55 (93.2%)
Positive history of red tide symptoms with exposure	37 (82.2%)
Current smoker	5 (8.5%)
Number hospitalized in ≥ 1 in past year from respiratory causes	11 (19.6%)

a Based on baseline questionnaire information.

b Predominantly β _2_ agonists.

**Table 3:** Self-reported symptoms and spirometry results for study participants preexposure and postexposure to beach.

	No red tide exposure		Red tide exposure	
Reported symptom	Preexposure = no Postexposure = yes (*n*)	Pre- vs. post-difference significance^a^	Preexposure = no Postexposure = yes (*n*)	Pre- vs. post-difference significance^a^
Respiratory				
Cough	9	0.44	15	0.01
Wheezing	4	0.74	7	0.03
Shortness of breath	7	0.56	8	0.06
Chest tightness	8	0.25	17	0.002
Other				
Throat irritation	5	0.56	12	0.02
Nasal congestion	6	0.76	12	0.25
Eye irritation	3	1.00	9	0.01
Headache	5	0.26	6	0.06
Itchy skin	1	0.32	1	0.56
Diarrhea	0	0	1	1.00

Spirometry value	Prebeach mean ± SD	Mean difference ± SD postexposure	Significance (*p*-Value)*^b^*	Prebeach mean ± SD	Mean difference ± SD postexposure	Significance (*p*-Value)*^b^*
FEV_1_	3.01 ± 0.88 L	21.0 ± 139.0 mL	0.24	3.03 ± 0.87 L	38.0 ± 118.0 mL	0.02
FVC	4.02 ± 1.07 L	2.0 ± 179.0 mL	0.93	4.04 ± 1.03 L	35.0 ± 176.0 mL	0.13
FEV_1_/FVC	75% ± 9%	0.6% ± 3%	0.09	75% ± 9%	0.3% ± 3%	0.48
FEF_25–75_	2.48 ± 1.19 L/sec	39.0 ± 332.0 mL/sec	0.36	2.53 ± 1.26 L/sec	95.0 ± 296.0 mL/sec	0.02
PEF	7.56 ± 2.02 L/sec	42.0 ± 693.0 mL/sec	0.64	7.59 ± 2.02 L/sec	81.0 ± 458.0 mL/sec	0.18

a McNemar’s test.

b Paired *t*-test.
